# Troponin Marker for Acute Coronary Occlusion and Patient Outcome Following Cardiac Arrest

**DOI:** 10.5811/westjem.2015.10.28346

**Published:** 2015-12-08

**Authors:** David A. Pearson, Catherine M. Wares, Katherine A. Mayer, Michael S. Runyon, Jonathan R. Studnek, Shana L. Ward, Kathi M. Kraft, Alan C. Heffner

**Affiliations:** * Carolinas Medical Center, Department of Emergency Medicine, Charlotte, North Carolina; † Mecklenburg EMS Agency, Charlotte, North Carolina; ‡ Carolinas Health Care System, Dickson Advanced Analytics Group, Charlotte, North Carolina; § Carolinas Medical Center, Department of Internal Medicine, Division of Critical Care Medicine, Charlotte, North Carolina

## Abstract

**Introduction:**

The utility of troponin as a marker for acute coronary occlusion and patient outcome after out-of-hospital cardiac arrest (OHCA) is unclear. We sought to determine whether initial or peak troponin was associated with percutaneous coronary intervention (PCI), OHCA survival or neurological outcome.

**Methods:**

Single-center retrospective-cohort study of OHCA patients treated in a comprehensive clinical pathway from November 2007 to October 2012. Troponin I levels were acquired at presentation, four and eight hours after arrest, and then per physician discretion. Cardiac catheterization was at the cardiologist’s discretion. Survival and outcome were determined at hospital discharge, with cerebral performance category score 1–2 defined as a good neurological outcome.

**Results:**

We enrolled 277 patients; 58% had a shockable rhythm, 44% survived, 41% good neurological outcome. Of the 107 (38%) patients who underwent cardiac catheterization, 30 (28%) had PCI. Initial ED troponin (median, ng/mL) was not different in patients requiring PCI vs no PCI (0.32 vs 0.09, p=0.06), although peak troponin was higher (4.19 versus 1.57, p=0.02). Of the 85 patients who underwent cardiac catheterization without STEMI (n=85), there was no difference in those who received PCI vs no PCI in initial troponin (0.22 vs 0.06, p=0.40) or peak troponin (2.58 vs 1.43, p=0.27). Regarding outcomes, there was no difference in initial troponin in survivors versus non-survivors (0.09 vs 0.22, p=0.11), or those with a good versus poor neurological outcome (0.09 vs 0.20, p=0.11). Likewise, there was no difference in peak troponin in survivors versus non-survivors (1.64 vs 1.23, p=0.07), or in those with a good versus poor neurological outcome (1.57 vs 1.26, p=0.14).

**Conclusion:**

In our single-center patient cohort, peak troponin, but not initial troponin, was associated with higher likelihood of PCI, while neither initial nor peak troponin were associated with survival or neurological outcome in OHCA patients.

## INTRODUCTION

More than 359,000 out-of-hospital cardiac arrests (OHCAs) occur each year in the U.S.^1^ For more than a decade, therapeutic hypothermia (TH) has been shown to improve survival with good neurological outcome.^2^–^4^ Beyond TH, early identification of the arrest etiology is another resuscitative priority, with acute coronary occlusion remaining a common cause.^5^ Clinical features and electrocardiogram (EKG) are poorly predictive of acute coronary occlusion in comatose patients after OHCA. Additionally, little is known about cardiac troponin as a marker for acute coronary occlusion and patient outcome in OHCA patients undergoing TH.

Current guidelines recommend immediate coronary angiography for suspected acute myocardial infarction (AMI) in patients successfully resuscitated after cardiac arrest.^6^ More specifically, guidelines encourage immediate angiography for OHCA patients with initial EKGs showing ST-elevation myocardial infarction (STEMI)^6^. A dilemma occurs, however, in that many patients resuscitated from cardiac arrest do not have ST-elevations on initial EKG, despite the possibility of a coronary occlusion.^7^ Thus, the challenge is identifying which non-STEMI patients have likely suffered cardiac arrest due to an acute coronary occlusion.

Troponin has been identified as a potential marker for acute coronary occlusion in the setting of cardiac arrest, as well as a potential marker for patient outcomes following cardiac arrest. Studies investigating various troponin assays, including newer high-sensitivity troponin T assays have shown mixed results.^8^,^9^ Additionally, few studies have investigated troponin’s association with survival, neurological outcome, and percutaneous coronary intervention (PCI), in the setting of TH.^10^ While previous studies have been conflicting on whether external defibrillation results in troponin elevation, recent studies using the high-sensitivity troponin T assay show external defibrillation can lead to an increased tro po nin.^11^,^12^

We sought to determine whether initial or peak cardiac troponin was associated with acute coronary occlusion, survival, or neurologic outcome in OHCA patients.

## METHODS

This was a retrospective cohort analysis on a prospectively collected post-cardiac arrest QI database. Patients were included for analysis if they were treated with TH in a comprehensive post-cardiac arrest clinical pathway known as Code Cool™, and were enrolled from November 2007 through October 2012. We enrolled all patients following admission to Carolinas Medical Center (CMC), an urban, 900-bed teaching hospital. Our center is a cardiac arrest receiving hospital with a network of 25 transferring hospitals in the region, as well as an STEMI receiving hospital. CMC is designated by the American Heart Association Mission: Lifeline® regional systems of care program, and is accredited by the Society of Chest Pain Centers. Sixty percent of our resuscitated cardiac arrest patients are brought directly to our cardiac arrest receiving hospital, while 40% are transferred, after initial resuscitation at a transferring hospital ED.

Patient inclusion and exclusion into our Code Cool™ protocol has been previously described.^13^ Briefly, resuscitated victims of out-of-hospital, non-traumatic cardiac arrest, with persistent coma (Glasgow Coma Scale [GCS] ≤8 and/or unable to follow verbal commands 15 minutes following ROSC) were eligible. All non-traumatic patients were eligible for the clinical TH pathway, at the discretion of the attending physician, regardless of initial arrest rhythm. Post-arrest care is standardized via protocol with patients cooled to 33°C for 24 hours then controlled rewarming at <0.5°C per hour, maintenance of mean arterial pressures >70mmHg via norepinephrine as needed, and avoidance of hyperventilation and hyperoxia. The Carolinas HealthCare System Institutional Review Board approved the study protocol.

We prospectively collected clinical data, including arrest type, treatment variables, and outcome, on consecutive patients with the use of a preformatted standard data collection tool using Utstein criteria. Survival and neurological outcomes were determined at the time of hospital discharge, with neurologic outcome being measured by the Pittsburgh cerebral performance category (CPC) scale. A “good neurological outcome” was defined as a CPC of 1 or 2.^14^ CPC 1 is defined as good cerebral performance and equates to patients that are conscious, alert, able to work, and might have mild neurologic or psychologic deficit. CPC 2 is defined as moderate cerebral disability and equates to patients that are conscious with sufficient cerebral function for independent activities of daily life and ability to work in a sheltered environment.

The primary outcome was the association of the initial or peak troponin with PCI. Acknowledging the time-sensitive nature of an acute coronary occlusion, the initial ED troponin was chosen as a potential marker for an acute coronary occlusion and the need for emergent coronary catheterization. However, as troponin elevations may go undetected if drawn less than six hours from an acute coronary occlusion, we chose to analyze peak hospitalization troponin as well. According to our standard clinical pathway, troponin I levels were acquired upon ED presentation, at four hours and eight hours after OHCA, and thereafter at physician discretion. Troponin I assays were performed using either the iSTAT platform or Abbott Architect Clinical Chemistry Analyzer (Abbott Diagnostics, Lake Forest, IL, USA), depending on standard laboratory practices of each institution. The decision to proceed to cardiac catheterization was made at the discretion of the cardiologist in accordance with predetermined institutional guidelines, which recommend emergent cardiac catheterization for survivors of OHCA with EKG findings of ST-elevations, age less than 75 years, and collapse to ROSC time less than 20 minutes. Patients not meeting these criteria were rapidly evaluated on a case-by-case basis by the cardiology team.

Secondary outcomes studied were the association between the initial and peak troponin levels with survival and good neurological outcome. We performed additional subgroup analysis on patients without STEMI to assess the association of initial and peak troponin with PCI, survival, and neurologic outcome.

For the statistical analysis, we assessed categorical variables with the chi-square or Fisher’s exact tests for small counts. T-tests and Wilcoxon rank-sum tests were used for continuous data, depending upon the distribution of the data. P-values less than 0.05 were considered statistically significant. We conducted all analyses using SAS statistical software version 9.2 (SAS Institute, Cary, NC).

## RESULTS

We screened 279 patients in our post-arrest TH database, excluded two patients with missing or unattainable data for witnessed arrest, initial rhythm, survival, neurological outcome, and cardiac catheterization, and analyzed the remaining 277 patients. Of the 277 patient studied, they had a median age 58 years (SD 14 years), 62% male, median time of arrest to ROSC of 21 minutes (SD 53 minutes), and initial shockable rhythm present in 58%. Demographics, arrest characteristics, EKG with STEMI, and troponins in all patients, as well as those with and without cardiac catheterization, are shown in Table 1. One hundred twenty-two patients (44%) survived to hospital discharge, 115 (41%) with good neurologic outcome. A total of 107 patients underwent cardiac catheterization with 22 STEMIs, 85 without STEMI, and 38 (36%) going emergent to cardiac catheterization.

We performed a primary analysis to assess whether initial or peak troponin level was associated with PCI. Of the 107 (38%) patients undergoing cardiac catheterization, the initial arrest rhythm in 91 (85%) was VT/VF versus 16 (15%) with asystole or PEA. Thirty out 107 patients (28%) had PCI and 15 of the 30 (50%) had a STEMI. Fifty-nine (55%) patients had their peak troponin before cardiac catheterization with a median time from ED arrival to peak troponin of 649 minutes. Forty-six (43%) patients had a cardiac catheterization within six hours, with the median time from hospital arrival to cardiac catheterization of 68 minutes. Although median initial troponin (IQR) was not significantly different in patients requiring PCI versus patients who did not, (0.32 [0.09–1.18] vs 0.09 [0.04–0.71)], p=0.06); median peak troponin (IQR) was higher in those patients who received PCI therapy (4.19 [1.56–7.53] vs 1.57 [0.52–5.44], p=0.02) (Table 2).

We performed further subgroup analysis on patients without STEMI. Eight-five patients out of 107 who underwent cardiac catheterization were without STEMI on ECG. There was no statistical difference in median initial or peak troponins in patients requiring PCI, survival, or neurological outcome (Table 3).

A secondary analysis was performed to assess whether initial ED troponin or peak hospitalization troponin were associated with survival and neurological outcome.

There was no difference in median (IQR) initial troponin in survivors versus non-survivors (0.09 [0.04–0.78] vs 0.22 [0.06–0.58], p=0.11). Likewise, there was no difference in median (IQR) peak troponin in survivors versus non-survivors (1.64 [0.54–6.39] vs 1.23 [0.29–4.85], p=0.07) (Table 4). With regards to neurologic outcome, there was no difference in median (IQR) initial ED troponin (ng/mL) in patients with a good versus poor neurological outcome (0.09 [0.04–0.78] vs 0.20 [0.06–0.61], p=0.11) and no difference in median (IQR) peak troponin (ng/mL) in patients with a good versus poor neurological outcome (1.57 [0.54–6.18] vs 1.26 [0.29–5.17], p=0.14) (Table 5).

## DISCUSSION

In our study population, peak troponin but not initial troponin levels were associated with PCI in our OHCA cohort who underwent a TH clinical pathway. However, in patients without STEMI, neither initial nor peak troponins were associated with patients receiving PCI. Additionally, initial and peak troponin levels were not associated with survival or neurological outcome.

Troponin elevation after cardiac arrest may be caused by several mechanisms including ischemic insult of arrest, direct effect of defibrillation, and coronary occlusion.^15^ The extent of ischemic insult following cardiac arrest is highly variable. Prior work has demonstrated higher troponin levels in patients with longer durations of resuscitation, and in patients with cardiogenic shock following ROSC.^16^

While previous studies have been conflicting on whether external defibrillation results in troponin elevation, newer studies using the high-sensitivity troponin T assays show that external defibrillation can lead to an increased troponin^8^,^9^ In another study, troponin elevation in implanted defibrillator discharges was an independent risk factor for mortality, but did not reliably differentiate those patients with AMI or acute coronary occlusion from tho se without.^17^ In studies using older troponin assays, elevated troponin levels did not reliably predict short-term outcome.^18^ Our study showed no significant difference in initial or peak troponin level among OHCA survivors versus non-survivors. Thus, based on our results, troponin does not appear to be useful for survival or neurologic prognostication.

The latest AHA guidelines encourage immediate coronary angiography for those with suspected AMI.^6^ Current STEMI guidelines recommend cardiac arrest patients with STEMI undergo emergent cardiac catheterization for potential PCI. Resuscitated patients after cardiac arrest without STEMI pose a challenge, as it is unclear which subgroup of these patients might benefit from cardiac catheterization.

Thus, the potential role of troponin as a biomarker to detect a recent coronary occlusion in OHCA has stirred interest. However, in a recent study, nearly all resuscitated OHCA patients, regardless of initial arrest rhythm, had a detectable troponin I, and most met biomarker guideline criteria for MI.^19^ Using standard normal troponin ranges in post-cardiac arrest to identify coronary occlusion appears to be of limited utility. With this in mind, researchers have attempted to determine if there was an optimal troponin threshold to detect a recent coronary occlusion in out-of-hospital arrest.^10^,^20^,^21^ From this work, a single initial ED troponin appears to have minimal utility in diagnosing or excluding MI after cardiac arrest. Even using the optimal cardiac troponin I threshold of 4.66 ng/mL to identify a recent coronary occlusion at admission, the sensitivity and specificity of this lab value is only 67% and 66% respectively.^10^ Another study in post-arrest patients revealed the optimal troponin cutoff of 2.5 ng/mL achieved a sensitivity and specificity of 72% and 75%, respectively, for the detection of a recent coronary occlusion.^20^

Given the uncertainty of troponin level relevance in OHCA, we sought to explore if troponin levels could be used as a marker for coronary occlusion in our population of patients undergoing TH. More specifically, we investigated the potential for initial ED troponin to predict which patients might benefit from emergent cardiac catheterization, as well as the utility of peak hospitalization troponin, to guide which patients might benefit from urgent cardiac catheterization.

Our study showed that peak troponin, but not initial troponin, was associated with PCI. Unfortunately, this has limited clinical utility because the myocardial damage is likely extensive when the peak troponin value is used to determine cardiac catheterization candidacy. Of the 107 patients who underwent cardiac catheterization, only 28% received PCI, of which 15 (50% of those receiving PCI) were patients with STEMI. Interestingly, excluding the STEMI patients among those chosen for cardiac catheterization, only 15 out of 85 (18%) received PCI. This suggests that in our cohort of patients without STEMI, either current strategies are suboptimal at determining who needs a cardiac catheterization, or that aggressively cardiac catheterizing OHCA patients may be of low yield. Unfortunately, initial ED troponin was not useful to identify those patients without STEMI that might benefit from emergent cardiac catheterization in our study. Given this finding, future work should explore if early serial troponins, such as the delta increase in troponin level, can be used as markers for acute coronary occlusion amendable to emergency coronary intervention.

Limitations of our study include a sample size that limits our ability to statistically discriminate small but potentially important outcome differences. The potential for unrecognized bias is also present given the non-randomized study design, and the fact that the inclusion of patients with non-shockable rhythms into the clinical pathway was at the discretion of the treating physician. Additionally, while all but one STEMI patient underwent emergent cardiac catheterization, patients without STEMI were evaluated on a case-by-case basis by our cardiology team to determine cardiac catheterization candidacy, with only 16 of 85 (19%) of patients without STEMI who underwent emergent cardiac catheterization. One major limitation in our study is that cardiologist were not blinded to the initial troponin levels and thus likely considered troponin level, in conjunction with age, demographic, and arrest characteristics, in their decision to perform angiography. Thus, selection bias is possible in the patient cohort that underwent angiography; however, this is less likely given the mean initial troponin were similar in patients undergoing versus those not undergoing cardiac catheterization (0.18 ng/mL vs 0.15 ng/mL, p=0.44). We also do not have data on the extent of the coronary lesions (i.e., findings of acute thrombus on cardiac catheterization) requiring PCI and thus we cannot draw any conclusions regarding acute coronary occlusion as the cause of the arrest based on PCI performance. Elevation of troponin levels can vary based on baseline patient characteristics, renal function, and presence of prior coronary artery disease, yet our database did not capture these baseline characteristics. We did not obtain the pre-PCI peak troponin nor post-PCI troponins during data extraction, which may have been more useful measurements over peak hospitalization troponin in retrospect, especially since PCI itself may cause troponin elevation. Regardless, our study showed that in the non-STEMI population where it is very challenging to identify those that might benefit by cardiac catheterization, we found no difference in peak troponins despite not further analyzing the sub-groups of pre- and post-PCI, and thus do not believe this analysis would provide additional clinically useful information. Our study was performed in an urban metropolitan hospital with relatively short transport times and may not be generalizable to regions with longer transport intervals. Our study population includes those selected as good candidates for an aggressive post-cardiac arrest resuscitative pathway, rather than the mandatory inclusion of all patients initially resuscitated from OHCA. This flexibility in the protocol potentially introduces selection bias, as the intervention is already being provided at the time of enrollment, and this may influence physicians to continue the TH protocol on the in-patient side, thereby including the patient in the data analysis. We believe this method is generalizable; however, as accrual is more reflective of clinical medicine outside of the research setting. The troponin I assays were performed on two different machines, namely the iSTAT and Abbott Architect platforms, which risks variability in the results obtained. Finally, although a logistic regression analysis could be performed to control for the multiple demographic and arrest variables, the authors felt this was of limited utility given the relatively small number of patients who underwent cardiac catheterization, and thus limits the strength on the association between troponin with PCI, survival, and neurological outcome.

## CONCLUSION

In our cohort of OHCA patients, peak troponin, though not initial troponin, was associated with need for PCI. When excluding STEMI patients, neither peak nor initial troponin was associated with need for PCI. Initial and peak troponin were also not associated with survival or neurological outcome. Future work should explore if early serial troponins can be used to detect acute coronary occlusion amendable to emergency coronary intervention.

## Figures and Tables

**Table 1:** Demographic, arrest characteristics, initial ECG, and troponins, and outcomes for overall cohort and in patients with and without cardiac catheterization.

	All patients (N=277)	Patients with cardiac catheterization (N=107)	Patients without catheterization (N=170)	p-value
	
	N (%)	N (%)	N (%)	
Age (Median, years)	58 (14)	58 (12)	58 (16)	0.96
Sex: Male	174 (62%)	76 (71%)	96 (56%)	0.02
Arrest interval (median (SD), minutes)	21 (53)	15 (13)	24 (68)	0.04
Initial rhythm				<0.0001
Shockable (VT/VF)	163 (58%)	91 (85%)	72 (43%)	
Non-shockable (PEA/asystole)	110 (39%)	16 (15%)	94 (55%)	
Unknown	4 (3%)	0 (0%)	4 (2%)	
Bystander CPR				0.10
Yes	178 (64%)	75 (70%)	103 (61%)	
No	77 (27%)	24 (22%)	53 (31%)	
Missing	24 (9%)	8 (8%)	14 (8%)	
Witnessed arrest*	233 (84%)	98 (92%)	135 (79%)	0.007
EKG findings				<0.0001
Without STEMI	254 (91%)	85 (79%)	169 (99%)	
STEMI	23 (8%)	22 (21%)	1 (1%)	
Initial troponin, mean (ng/mL)	0.18	0.18	0.15	0.44
Peak troponin, mean (ng/mL)	1.71	2.02	0.97	0.01
Survival*	122 (44%)	86 (80%)	36 (21%)	<0.0001
Neurologic outcome (CPC)^*^				<0.0001
CPC 1–2 (Good neuro outcome)	115 (41%)	23 (22%)	31 (18%)	
3–5 (Poor neuro outcome)	162 (58%)	84 (78%)	139 (82%)	

*VT,* ventricular tachycardia; *VF,* ventricular fibrillation; *PEA,* pulseless electrical activity; *CPR,* cardiopulmonary resuscitation; *EKG,* electrocardiogram; *STEMI,* ST segment elevation myocardial infarction; *CPC,* cerebral performance category

**Table 2:** Association with initial and peak troponin with percutaneous coronary intervention (PCI).

	PCI	No PCI	p-value
Patients	n=30 (28%)	n=77 (72%)	
Initial troponin, ng/mL (mean, SD)	0.32 (0.09–1.18)	0.09 (0.04–0.71)	0.06
Peak troponin, ng/mL (mean, SD)	4.19 (1.56–7.53)	1.57 (0.52–5.44)	0.02

**Table 3:** Subgroup analysis of patients with cardiac catheterization and without ST segment elevation myocardial infarction (n=85).

	Initial troponin, median (IQR)	p-value	Peak troponin, median (IQR)	p-value
Survive		0.14		0.86
Yes	0.06 (0.03 – 0.55)		1.57 (0.58 – 6.39)	
No	0.29 (0.19 – 0.68)		1.86 (0.69 – 4.60)	
Neurologic outcome (CPC)		0.07		0.76
Good (CPC 1–2)	0.06 (0.03 – 0.53)		1.57 (0.55 – 6.29)	
Bad (CPC 3–5)	0.30 (0.21 – 0.71)		2.02 (0.75 – 4.68)	
PCI				
Yes	0.22 (0.06 – 0.5)	0.40	2.58 (1.30 – 6.39)	0.27
No	0.06 (0.03 – 0.71)		1.43 (0.52 – 5.06)	

*CPC,* cerebral performance category; *PCI,* percutaneous coronary intervention

**Table 4:** Association of initial and peak troponin with survival.

	Survivors	Non-survivors	p-value
Patients	n=122 (44%)	n=155 (55%)	
Initial troponin, ng/mL (mean, SD)	0.09 (0.04–0.78)	0.22 (0.06–0.58)	0.11
Peak troponin, ng/mL (mean, SD)	1.64 (0.54–6.39)	1.23 (0.29–4.85)	0.07

**Table 5:** Association of initial and peak troponin with neurologic outcome.

	CPC 1/2	CPC 3/4/5	p-value
Patients	n=115 (41%)	n=162 (58%)	
Initial troponin, ng/mL (mean, SD)	0.09 (0.04–0.78)	0.20 (0.06–0.61)	0.11
Peak troponin, ng/mL (mean, SD)	1.57 (0.54–6.18)	1.26 (0.29–5.17)	0.14

*CPC,* cerebral performance category

## References

[1] Go AS, Mozaffarian D, Roger VL (2014). Heart disease and stroke statistics--2014 update: a report from the American Heart Association. Circulation.

[2] The Hypothermia after Cardiac Arrest Study Group (2002). Mild therapeutic hypothermia to improve the neurologic outcome after cardiac arrest. N Engl J Med.

[3] Bernard SA, Gray TW, Buist MD (2002). Treatment of comatose survivors of out-of-hospital cardiac arrest with induced hypothermia. N Engl J Med.

[4] Nielsen N, Wetterslev J, Cronberg T (2013). Targeted temperature management at 33 degrees C versus 36 degrees C after cardiac arrest. N Engl J Med.

[5] Peberdy MA, Callaway CW, Neumar RW (2010). Part 9: post-cardiac arrest care: 2010 American Heart Association Guidelines for Cardiopulmonary Resuscitation and Emergency Cardiovascular Care. Circulation.

[6] O’Gara PT, Kushner FG, Ascheim DD (2013). 2013 ACCF/AHA guideline for the management of ST-elevation myocardial infarction: a report of the American College of Cardiology Foundation/American Heart Association Task Force on Practice Guidelines. J Am Coll Cardiol.

[7] Dumas F, Cariou A, Manzo-Silberman S (2010). Immediate percutaneous coronary intervention is associated with better survival after out-of-hospital cardiac arrest: insights from the PROCAT (Parisian Region Out of hospital Cardiac Arrest) registry. Circ Cardiovasc Interv.

[8] Geri G, Mongardon N, Dumas F (2013). Diagnosis performance of high sensitivity troponin assay in out-of-hospital cardiac arrest patients. Int J Cardiol.

[9] Lund M, French JK, Johnson RN (2000). Serum troponins T and I after elective cardioversion. Eur Heart J.

[10] Dumas F, Manzo-Silberman S, Fichet J (2012). Can early cardiac troponin I measurement help to predict recent coronary occlusion in out-of-hospital cardiac arrest survivors?. Crit Care Med.

[11] Huang J, Walcott GP, Ruse RB (2012). Ascending-ramp biphasic waveform has a lower defibrillation threshold and releases less troponin I than a truncated exponential biphasic waveform. Circulation.

[12] Piechota W, Gielerak G, Ryczek R (2007). Cardiac troponin I after external electrical cardioversion for atrial fibrillation as a marker of myocardial injury--a preliminary report. Kardiol Pol.

[13] Heffner AC, Pearson DA, Nussbaum ML (2012). Regionalization of post-cardiac arrest care: implementation of a cardiac resuscitation center. Am Heart J.

[14] Benz-Woerner J, Delodder F, Benz R (2012). Body temperature regulation and outcome after cardiac arrest and therapeutic hypothermia. Resuscitation.

[15] Allan JJ, Feld RD, Russell AA (1997). Cardiac troponin I levels are normal or minimally elevated after transthoracic cardioversion. J Am Coll Cardiol.

[16] Mullner M, Oschatz E, Sterz F (1998). The influence of chest compressions and external defibrillation on the release of creatine kinase-MB and cardiac troponin T in patients resuscitated from out-of-hospital cardiac arrest. Resuscitation.

[17] Blendea D, Blendea M, Banker J (2009). Troponin T elevation after implanted defibrillator discharge predicts survival. Heart.

[18] Lai CS, Hostler D, D’Cruz BJ (2004). Prevalence of troponin-T elevation during out-of-hospital cardiac arrest. Am J Cardiol.

[19] Kontos MC, de Lemos JA, Ou FS (2010). Troponin-positive, MB-negative patients with non-ST-elevation myocardial infarction: An undertreated but high-risk patient group: Results from the National Cardiovascular Data Registry Acute Coronary Treatment and Intervention Outcomes Network-Get With The Guidelines (NCDR ACTION-GWTG) Registry. Am Heart J.

[20] Voicu S, Sideris G, Deye N (2012). Role of cardiac troponin in the diagnosis of acute myocardial infarction in comatose patients resuscitated from out-of-hospital cardiac arrest. Resuscitation.

